# Acute Stress-Induced Epigenetic Modulations and Their Potential Protective Role Toward Depression

**DOI:** 10.3389/fnmol.2018.00184

**Published:** 2018-05-31

**Authors:** Francesco Rusconi, Elena Battaglioli

**Affiliations:** ^1^Department of Medical Biotechnologies and Translational Medicine, University of Milan Via Fratelli Cervi, Segrate, Italy; ^2^CNR Institute of Neuroscience Via Vanvitelli, Milan, Italy

**Keywords:** epigenetics mechanisms of plasticity, depressive disorder, stress, psychological, LSD1, hippocampus

## Abstract

Psychiatric disorders entail maladaptive processes impairing individuals’ ability to appropriately interface with environment. Among them, depression is characterized by diverse debilitating symptoms including hopelessness and anhedonia, dramatically impacting the propensity to live a social and active life and seriously affecting working capability. Relevantly, besides genetic predisposition, foremost risk factors are stress-related, such as experiencing chronic psychosocial stress—including bullying, mobbing and abuse—, and undergoing economic crisis or chronic illnesses. In the last few years the field of epigenetics promised to understand core mechanisms of gene-environment crosstalk, contributing to get into pathogenic processes of many disorders highly influenced by stressful life conditions. However, still very little is known about mechanisms that tune gene expression to adapt to the external milieu. In this Perspective article, we discuss a set of protective, functionally convergent epigenetic processes induced by acute stress in the rodent hippocampus and devoted to the negative modulation of stress-induced immediate early genes (IEGs) transcription, hindering stress-driven morphostructural modifications of corticolimbic circuitry. We also suggest that chronic stress damaging protective epigenetic mechanisms, could bias the functional trajectory of stress-induced neuronal morphostructural modification from adaptive to maladaptive, contributing to the onset of depression in vulnerable individuals. A better understanding of the epigenetic response to stress will be pivotal to new avenues of therapeutic intervention to treat depression, especially in light of limited efficacy of available antidepressant drugs.

## Introduction

Molecular psychiatry mainly recognizes three typologies of stressful events, namely positive stress, tolerable stress and toxic stress (McEwen, 2017). “Positive” stress (also known as eustress; Selye, 1998) entails reward-associated paradigms including whatever hard paths to meet our life expectations (job promotions and achievements in general). On the other hand, we can recognize two typologies of negative stress (also known as distress; Selye, 1998), namely “tolerable” and “toxic” stress. “Tolerable” stress has to do with negative experiences, prototypically represented by loss of beloved persons, but also related to personal, economic or health crisis (McEwen, 2017). Regardless how hard this kind of stress can be, it is identified by those experiences that most people have the mental instruments to cope with, also thanks to supportive relationships (McEwen et al., 2015). However, behavioral outcome of negative contingencies can vary in an individual-specific manner accordingly to the set of previous life experiences and with respect to genetic background in the frame of genotype × environment interactions (GxE; de Kloet et al., 2005; Caspi and Moffitt, 2006; Joëls and Baram, 2009; McEwen, 2012; Sun et al., 2013; Calhoon and Tye, 2015). Indeed, also a negative stress theoretically predictable as tolerable could lead to psychiatric issues in a subset of the general population referred to as stress-vulnerable individuals. Beside the intensity and aversiveness of the stressful stimuli, chronic reiteration or duration of a negative experience can change their outcome often leaving long-term mood effects (McEwen, 2003; Cohen et al., 2007). “Toxic” stress is represented by those experiences featuring recurrent parameters of unpredictability and inescapability (terrorist attacks, earthquake, military operations; McEwen, 2017). This form of stress is considered detrimental for the majority of population and can lead to post traumatic stress disorder (PTSD) and other neuropsychiatric issues (McEwen, 2005; Nagy et al., 2017). Nonetheless, also in case of toxic stress, there are individuals displaying resiliency (McEwen et al., 2015). Consistently, a relevant open question in neurobiology of stress response is related to molecular underpinnings of vulnerability or resiliency. Tolerable stress with a positive behavioral outcome can be modeled in rodents using a single acute stressful paradigm experienced by naïve mice or rats, since such a challenge does not elicit long-term behavioral effects on a cognitive or emotional point of view. On the contrary, chronic administration of the same forms of stress can precipitate mood and cognition-related issues in a subset of susceptible individuals (Golden et al., 2011; McEwen et al., 2015).

## Pathways of Stress Response

The most studied pathway of stress response is referred to as the hypothalamic-pituitary-adrenal (HPA) axis. This system allows environmental adaptation via a complex interplay between two sets of processes acting at the molecular and cellular level and influencing behavioral responses. The first set underlies the arousal phase of stress response (*primary response*), which includes reactions leading to the required wakefulness to respond to threat, invigorating physical strength and cognitive acuity (Davis et al., 2003). The second set is related to stress termination and more in general to homeostatic processes in the body aimed at restraining excessive reactions (*secondary response*). Glucocorticoid hormone is an important final effector of stress signals mainly involved in HPA axis homeostasis and feedback (Cohen et al., 2012). Stress termination is largely operated at the hippocampal level, brain area that is involved in stress-response, and that expresses a high level of glucocorticoid receptors. Besides HPA axis, a core process selected by evolution to survive to environmental changes, other systems contribute to cope with stress through a fine-tuning of glutamate response. A well-known pathway that helps responding to stress via glutamate signaling regulation is the endocannabinoid system (ECS). In this case, the *primary response* is represented by stress-induced glutamate release in brain areas that are activated by stress, which in turn promotes through ECS, depolarization-induced suppression of excitation (DSE) as the *secondary response*. ECS is highly effective in restraining the excitotoxic consequences of stress-induced glutamate, contrasting toxic behavioral correlates of environmental stress (Lutz et al., 2015; Morena et al., 2016).

A single stress event induces temporary activation of the stress-response machinery, and since they are not associated to long-lasting behavioral alterations, related modifications on neuronal physiology can be defined as adaptive (Hunter et al., 2009, 2012; Rusconi et al., 2016; Saunderson et al., 2016; McEwen, 2017). Interestingly, it is generally accepted that psychopathology must result from a failure in a correct functionality of HPA axis and ECS (McEwen, 2003; Weaver et al., 2004; Lutz et al., 2015; McEwen et al., 2015; Morena et al., 2016). In particular, excessive or reiterated engagement of stress-coping molecular mechanisms (including but not limited to the action of stress hormones) in a chronic manner, could lead to allostatic overload via desensitization of stress response pathways (Joëls and Baram, 2009; McEwen, 2017).

In this Perspective article, we shed new light on a further mechanism of stress-response based on epigenetic modifications of gene expression that cooperates at the nuclear level with HPA axis and ECS. We further emphasize the relevance of these adaptive mechanisms in response to acute stress as their impairment might represent a proxy for the onset of stress-related psychopathology (Borrelli et al., 2008; McEwen, 2012; Nestler, 2014; Nagy et al., 2017).

## Stress Impacts Neuronal Connectivity and Plasticity

Stress induces specific brain plasticity-modifying transcriptional programs in corticolimbic circuitry including the medial prefrontal cortex (mPFC), the ventral hippocampus (vHIP) and the amygdala (Felix-Ortiz et al., 2013; Janak and Tye, 2015; Laine et al., 2017). In these areas, behavioral stress has been recognized as a powerful modifier of neuronal structural plasticity (McEwen et al., 2012; Chattarji et al., 2015). Either acute or chronic stress can transiently or stably modify dendritic spine density and arborization (Golden et al., 2013; Maras et al., 2014; Chattarji et al., 2015; Janak and Tye, 2015), suggesting that adaptive structural modification of corticolimbic areas can also correspond to a neutral behavioral response. The magnitude and direction of stress-mediated neuroplastic remodeling vary according to the peculiar structure observed, generally increasing amygdalar neuroplasticity (enhancing fear reactions) and decreasing hippocampal and prefrontal cortex functionality (leading to impaired control over affective manifestation; McEwen et al., 2012; Felix-Ortiz et al., 2013; Chattarji et al., 2015). This bidirectional corticolimbic unbalance underlies aberrant top-down inhibition of the limbic system, a core symptom of neuropsychiatric disorders (Martin et al., 2009; Franklin et al., 2012; Calhoon and Tye, 2015).

## Acute Stress Elicits Adaptive Stress-Coping Epigenetic Mechanisms in the Hippocampus

Neurons are able to modify synaptic activity in response to environmental inputs—including stress—through epigenetic modifications allowing to finely and steadily perturb gene expression (Borrelli et al., 2008). Stress can engrave chromatin with a peculiar alphabet, made up of histone post-translational modifications (PTMs) and DNA methylation (Tsankova et al., 2006; Borrelli et al., 2008). The study of epigenetic modifications—ultimately shaping the intimacy of DNA-histone interactions regulating the accessibility and processivity of basal transcription machinery—operated by different sources of stress in the brain has just begun, and much more work is required to clarify the typology and relative behavioral relevance of specific epigenetic modulations.

**Figure 1:**
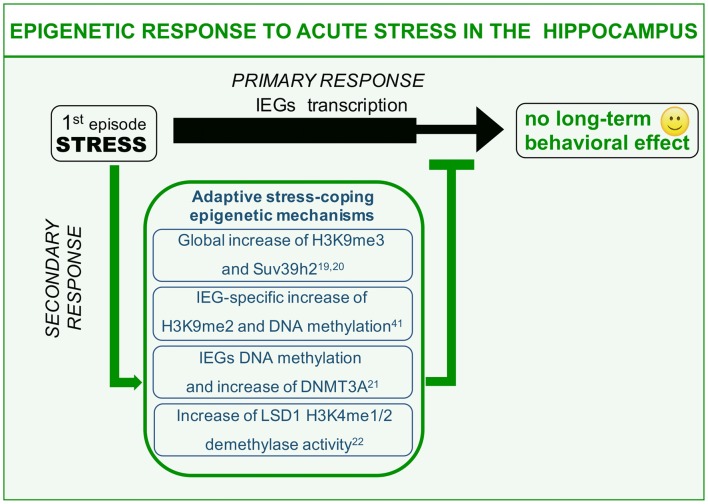
Epigenetic mechanisms of acute stress allostasis. Tolerable stress with a positive behavioral outcome elicits plasticity-gene transcription in the hippocampus instrumental to memorizing threat-related aspects of the negative experience with a protective valence. Meanwhile a set of epigenetic mechanisms buffer stress-induced transcription retaining its functional outcome within adaptive range. These mechanisms include global increase of H3K9me3 repressive histone mark, increased levels of DNA methyltransferase Dnmt3a as well as its association to the Immediate Early Genes (IEGs) promoters and increased Lysine-Specific Demethylase 1 (LSD1)-related repressive potential towards the same gene targets. This set of acute stress-induced epigenetic modifications contribute to counteract long-term behavioral effects on a cognitive or emotional point of view.

We here describe different examples of epigenetic processes that occur in response to acute “negative stress.” Immediate response to stress has been widely reported to induce glutamate-driven MAPK kinase pathway activation and consequent transient wave of plasticity-gene transcription including the immediate early genes (IEGs; *primary* *response*, Chwang et al., 2006; Gutièrrez-Mecinas et al., 2011; Figure 1). IEGs transcription should be instrumental to the balanced memorization of stressful event aimed at engaging a protective behavioral response against similar threatening situations, a response that however, does not have to excessively invigorate fear and anxiety. This can be achieved thanks to a delayed set of stress response mechanisms (*secondary responses*) that has to do with: (1) switching off immediate early transcriptional induction; and (2) increasing the threshold of IEGs transcriptional activation in the same circuitry for a limited time window (Figure 1). This second point, albeit observed, has never been clearly formalized on a functional point of view. Here, we will discuss examples of stress-induced *secondary responses* sharing a common epigenetic nature and predicted to be instrumental to adaptive molecular stress response.

### Global Increase of H3K9me3 and Suv39h2

It has been shown that acute restraint stress elicits significant global increase of repressive histone mark H3K9me3, together with increased levels of specific methyltransferase Suv39h2 (Hunter et al., 2009, 2012). These modulations represent hippocampus-specific *secondary response* at the level of CA1 and DG sub area, witnessing interplay between stress and gene expression. It is worth noting that chronic restraint stress administered for 7 days does not entail any modification of H3K9me3 levels in the same areas (Hunter et al., 2009). These data suggest that H3K9me3 global increase after acute stress might represent an allostatic mechanism that can be triggered only by a limited number of stressful events of the same nature. It is conceivable to hypothesize that globally increased gene repression (H3K9me3, heterochromatin formation) is related to adaptive acute stress-coping strategies aimed at pausing gene transcription to buffer acquired neuroplasticity. Moreover, this *secondary response* may be diminished or degraded by chronic stressful experiences (Hunter et al., 2009). Interestingly fluoxetine—a selective serotonin reuptake inhibitor (SSRI) commonly used as antidepressant—is highly effective in restoring global H3K9me3 increase during chronic stress. Relevantly, fluoxetine has also been reportedly shown to interfere with chronic stress-induced structural and behavioral correlates (Magariños et al., 1999; Czéh et al., 2007; Bessa et al., 2009). Hence, it is plausible that H3K9me3 increase upon SSRI treatment is related to the antidepressant potential of this drug. In other words, one of the effects of fluoxetine is to reinstate an allostatic process in response to chronic stress that is normally restricted to acute stress. Thus, to restore the chronic stress corrupted epigenetic-based *secondary response*, could represent a strategy to counteract toxic behavioral effects of repeated sessions of environmental stress.

### IEGs-Specific Increase of H3K9me2 and DNA Methylation

The foot shock paradigm, widely used to assess associative memory in rodents, also represents a highly negative experience scored as able to strongly induce molecular mechanisms of stress response in the hippocampus (Schöner et al., 2017). This paradigm can reproduce some of the core symptoms of PTSD including avoidance and anxiety behavior (Schöner et al., 2017). Interestingly, foot shock paradigm induces in the hippocampus of stressed animals IEGs transcription as *primary response*, which is supported by increased levels of the euchromatin-associated histone mark H3K4me3 at the level of the IEG *egr1* (Gupta et al., 2010). Such an activity-dependent transcription is instrumental to promote stress-plasticity as a form of fear-related memory of the negative experience. Meanwhile, a seemingly concomitant *secondary response* triggers increased H3K9me2 levels, a chromatin modification related to gene silencing. Moreover, also increased methylation at multiple CpGs at the level of *egr1* promoter region (Gupta et al., 2010) can be scored. Interestingly, treating mice with the HDAC inhibitor sodium butyrate before the stressful paradigm, along with significantly decreasing the global level of H3K9me2 methylation, also worsen the phenotypic read-out of foot shock, increasing the freezing behavior (Gupta et al., 2010). A possible interpretation of these evidences can be related to adaptive stress-coping strategies. In particular, concomitant induction of permissive (H3K4me3) and inhibitory (H3K9me2 and DNA methylation) chromatin modifications in the frame of a tight *egr1* transcriptional control can allow the perfect expression balance of plasticity genes, leading to a correct and protective memorization of the dangerous context, preventing in the meantime exaggerated behavioral arousals to future homotypic stressful experiences.

### IEGs-Specific Increase of DNA Methylation and Dnmt3a

We learned from the previous example that not only histone, but also DNA methylation at specific CpGs plays an important role in the behavioral responses to stressful situations. Recently, it has been shown that promoting DNA methylation of *c-fos* and *egr1* promoters via S-adenosyl methionine (SAM) administration, the endogenous methyl-donor for DNA methylation, it is possible to significantly hamper the consolidation of immobility behavior after forced swim (FS) paradigm in rats (Saunderson et al., 2016). Immobility behavior represents a phenotypical read-out of stress response whose interpretation is debated, being reported as either adaptive (Trollope et al., 2012), i.e., to conserve energies to survive, or depressive (Ramaker and Dulawa, 2017), in the sense that the animal refuses to make all possible attempts to exit the water. A first FS training induces IEG transcriptional activation as a *primary response* to stress, together with a permissive DNA demethylation at specific CpGs in *c-fos* and *egr1* promoters (Saunderson et al., 2016). These pro-transcriptional epigenetic modifications again are devoted to memorization of the stressful experience. Interestingly, in the same time window, the authors also observed an apparently paradoxical increase of DNA Methyl Transferase 3a (Dnmt3a) at both the transcriptional level and in terms of chromatin association to IEGs promoters. As expected, in a second FS test (re-test) animals significantly increase their immobility time, representing this phenotype a modified behavioral readout (cognitive and emotional), which depends on the first stress exposure in the frame of a behavioral sensitization. Interestingly, administration of SAM before the first stressful paradigm, blocks the behavioral outcome of the re-test, leaving immobility time unaltered. This is due to the fact that SAM, favoring the activity of physiologically increased Dnmt3a via increased substrate concentration, counteracts IEG transactivation in response to the first FS test, thus blocking consolidation of stress-induced plasticity, and reflecting on a decreased behavioral arousal in response to the second homotypic stress. In other words, stress-increased Dnmt3a repressive activity towards IEGs should represent another example of adaptive acute stress-coping process based on transcriptional constrain of plasticity genes in the hippocampus.

### Increase of LSD1 H3K4me1/2 Demethylase Activity

Another example of epigenetic modification elicited by acute stress involves the chromatin modifier Lysine-Specific Demethylase 1 (LSD1), also known as KDM1A, a transcriptional corepressor responsible for demethylation of histone H3K4me1/2 (Shi et al., 2004). Recently it has been reported that in the hippocampus, in response to a psychosocial stress paradigm, the social defeat stress (SDS), LSD1 repressive potential is increased following a single session of stress. In particular it was unveiled that LSD1 increase is the results of a transient reduction of neuroLSD1, a dominant negative LSD1 isoform unable to repress transcription, via neuro-specific alternative splicing mechanism (Wang et al., 2015; Rusconi et al., 2016). LSD1 and neuroLSD1, in association with the transcription factor SRF, control transcriptional proneness of the IEGs (Toffolo et al., 2014; Wang et al., 2015; Rusconi et al., 2016). Therefore, reported modification of the balance between the two isoforms in response to an acute stress, should negatively impact IEG transcription. Consistently, in a genetic model of neuroLSD1 haploinsufficiency (neuroLSD1^HET^)—mimicking the above described stress-induced neuroLSD1 decrease and increase of LSD1—stress-evoked transactivation of* c-fos* and *egr1* genes in the hippocampus is impaired (Rusconi et al., 2016). Interestingly, neuroLSD1^KO^ and heterozygous mice are also characterized by a very peculiar phenotype, a low anxiety-like behavior (Rusconi et al., 2016). Given that stress is highly effective in increasing the level of anxiety, the research group proposed that LSD1 mediated occlusion of stress-induced IEG transcription, must represent a *secondary epigenetic response* with adaptive meaning aimed at buffering excessive consolidation of stress plasticity in terms of anxiety (Rusconi et al., 2016, 2017). Relevantly, low anxiety of neuroLSD1 mutant mice can be restored to wild type levels enhancing IEG expression through administration of class I HDAC inhibitor suberoylanilide hydroxamic acid (SAHA; Rusconi et al., 2016). These results indicate a role for LSD1 in controlling IEGs expression in response to acute stress, participating together with the other epigenetic modifiers to homeostatic control of stress-response.

It is worth mentioning that although many brain areas participate to stress response (as described above) to the best of our knowledge, all the examples of epigenetic modulation following acute stress focused on the hippocampus as the brain area of interest. This does not mean that similar mechanisms cannot be triggered in other areas. However, described data further support an important role for hippocampus in stress adaptation.

The four above described examples can be clustered into a new category of protective stress response mechanisms collectively referred to as epigenetic stress response (ESR).

## Discussion

**Figure 2:**
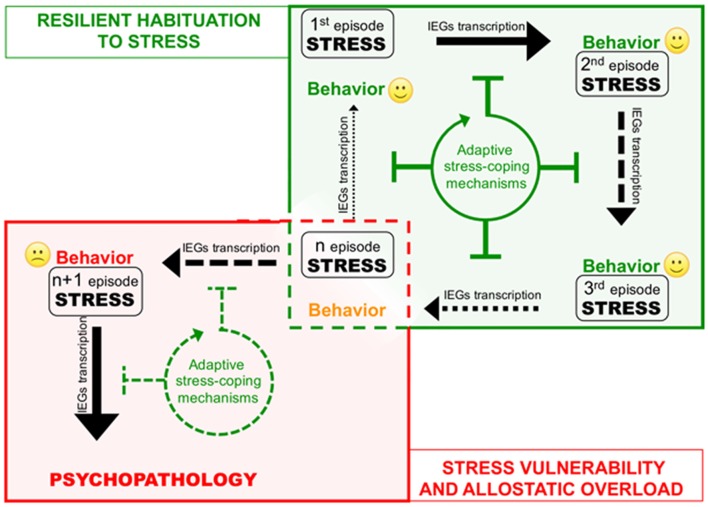
Corruption of epigenetic coping strategies (allostatic epigenetic overload) upon chronic stress reiteration might represent a cue to the onset of stress-related psychopathology. Consequential administration of homotypic stressful events (from stress 1 to n) induces plasticity-related transcription indicated by black arrows of different thickness depending on the transcription rate. Each stress event engages epigenetic allostatic mechanisms aimed at buffering stress-induced transcription and participating to stress habituation in resilient individuals. Stress habituation consists in a progressive reduction of the transcriptional response to stress (green box). However (red box), in vulnerable individuals, after a number of stressful events (n episode), chronic overuse of this protection system can lead to disruption of allostatic epigenetic processes. Loss of adaptive stress-coping mechanisms entails increasing stress-evoked transcription and consequent neuroplastic modifications precipitating psychopathology.

The cases described above shed new light on the existence of a previously unrecognized layer of stress response mechanisms the ESR. We retrieved and conceptually linked from the literature a set of epigenetic modifications occurring to control stress-induced transcription, representing novel examples of homeostatic processes elicited in rodent models by different environmental stressful events. Interestingly, in three out of four sets of data described, the IEGs were included among those genes that show negative epigenetic transcriptional modulation as a *secondary response* to environmental stress. Increase of DNMT3a (Saunderson et al., 2016), LSD1 (Rusconi et al., 2016) and Suv39H2 (Hunter et al., 2012), together with global increase of H3K9me2/3 (Hunter et al., 2009; Gupta et al., 2010) in the hippocampus in response to a single stress could represent concerted and functionally converging mechanisms aimed at decreasing IEGs stimulus-induced expression in response to a further homotypic stress. Given IEGs involvement in memory trace formation, reducing their transcriptional responsiveness via modification of promoters’ chromatin structure might associatively influence the emotional response to another similar experience, possibly participating to stress habituation (Figure 2). Epigenetic modifications have the intrinsic feature to last longer than a given stimulus *per se*, representing initial, chromatin encoded early step of memorization also in case of stressful events (Sweatt, 2016). However, this form of chromatin-based memory has to be transient, likewise all other circuitry modulations that concur to allostatic stress-response.

Acute stress is able to induce molecular modifications at the chromatin, transcriptional, structural and circuitry levels (Sun et al., 2013). However, acute stressful insults do not usually lead to long-term issues on a behavioral point of view (except for what concerns PTSD-inducing stimuli; Shvil et al., 2013). Therefore, it should be possible to hypothesize that, when the stress is tolerable, all different stress response-related allostatic mechanisms (HPA axis, ECS and ESR) represent important examples of adaptive molecular processes aimed at a correct interpretation and balanced memorization of stressful experiences (Lupien et al., 2009; Lutz et al., 2015; Rusconi et al., 2017).

On the other hand, excessive exposure to stress such as in case of chronic stress can cause desensitization and/or deterioration of the same allostatic pathways engaged by acute stress contributing to psychopatology (de Kloet et al., 2005). For instance, it is well known how chronic activation of the HPA axis leads to a deregulation of inflammation control in chronically stressed individuals (Cohen et al., 2012). A similar deterioration occurs at the level of the ECS, where CB1 desensitization blocking ECS-mediated buffering of stress-induced glutamate release and fosters the negative behavioral effects of stress (Lutz et al., 2015; Morena et al., 2016). In this Perspective article, we propose that chronic stress fosters the pathogenesis of stress-related depression also via disruption of epigenetic mechanisms of stress response. Consistently, in the first example reported above, chronic stress—contrary to acute—does not elicit protective epigenetic processes such as global increase of H3K9me3 levels in the hippocampus (Hunter et al., 2009, 2012). It would be interesting to understand whether also the acute stress-related molecular mechanisms underlying: (i) increase of LSD1-mediated H3K4me1/2 demethylase activity; and (ii) Dnmt3a upregulation and recruitment to IEG promoters, can be similarly less efficiently engaged upon chronic stress (Figure 2). In particular, we support the hypothesis that loss of epigenetic control over IEGs transcription in the hippocampus along with chronic stress, allowing exaggerated plasticity-related transcription, leads to vulnerability-associated corruption of corticolimbic circuitry. This hypothesis is supported by a further set of data demonstrating that IEGs increase in the vHIP, is related to chronic stress vulnerability (Bagot et al., 2015). Relevantly, in the same stress vulnerable mice, optogenetically reducing neuroplasticity by inducing LTD in the vHIP-NAc circuit, represents an effective treatment against the pro-depressive traits induced by chronic SDS (Bagot et al., 2015).

In conclusion, the novel described mechanisms of ESR that we propose to be protective against depression via tight control towards stress-induced IEGs transcription, together with the notion that in models of stress vulnerability IEGs are stably overexpressed (Bagot et al., 2015), clearly indicates an urgency to further elucidate ESR with the ultimate goal to understand the molecular basis of stress-induced depression, opening new avenues of interventions.

## Author Contributions

FR and EB conceived the idea and wrote the manuscript.

## Conflict of Interest Statement

The authors declare that the research was conducted in the absence of any commercial or financial relationships that could be construed as a potential conflict of interest.
